# A large scale expression study associates uc.283-plus lncRNA with pluripotent stem cells and human glioma

**DOI:** 10.1186/s13073-014-0076-4

**Published:** 2014-10-02

**Authors:** Marco Galasso, Paola Dama, Maurizio Previati, Sukhinder Sandhu, Jeff Palatini, Vincenzo Coppola, Sarah Warner, Maria E Sana, Riccardo Zanella, Ramzey Abujarour, Caroline Desponts, Michael A Teitell, Ramiro Garzon, George Calin, Carlo M Croce, Stefano Volinia

**Affiliations:** Biosystems Analysis, LTTA, Department of Morphology, Surgery and Experimental Medicine, Università degli Studi, Via Fossato di Mortara, 70, Ferrara, 44123 Italy; Comprehensive Cancer Center, Wexner Medical Center, and Biomedical Informatics, Ohio State University, Columbus, OH 43210 USA; Biomedical Informatics, Ohio State University, Columbus, OH 43210 USA; Experimental Therapeutics & Cancer Genetics, MD Anderson Cancer Center, Houston, TX 77030 USA; Fate Therapeutics, 3535 General Atomics Ct, San Diego, CA 92121 USA; Department of Chemistry, The Scripps Research Institute, 10550 North Torrey Pines Road, La Jolla, CA 92037 USA; Department of Pathology & Laboratory Medicine, David Geffen School of Medicine at UCLA, Los Angeles, CA 90095 USA

## Abstract

**Background:**

There are 481 ultra-conserved regions (UCRs) longer than 200 bases in the genomes of human, mouse and rat. These DNA sequences are absolutely conserved and show 100% identity with no insertions or deletions. About half of these UCRs are reported as transcribed and many correspond to long non-coding RNAs (lncRNAs).

**Methods:**

We used custom microarrays with 962 probes representing sense and antisense sequences for the 481 UCRs to examine their expression across 374 normal samples from 46 different tissues and 510 samples representing 10 different types of cancer. The expression in embryonic stem cells of selected UCRs was validated by real time PCR.

**Results:**

We identified tissue selective UCRs and studied UCRs in embryonic and induced pluripotent stem cells. Among the normal tissues, the uc.283 lncRNA was highly specific for pluripotent stem cells. Intriguingly, the uc.283-plus lncRNA was highly expressed in some solid cancers, particularly in one of the most untreatable types, glioma.

**Conclusion:**

Our results suggest that uc.283-plus lncRNA might have a role in pluripotency of stem cells and in the biology of glioma.

**Electronic supplementary material:**

The online version of this article (doi:10.1186/s13073-014-0076-4) contains supplementary material, which is available to authorized users.

## Background

Long non-coding RNAs (lncRNAs) are involved in many biological processes [1]. Transcribed ultra-conserved regions (T-UCRs) are a large portion of the so-called ultra-conserved regions (UCRs). The 'ultra-conserved' term was originally proposed for genomic regions longer than 200 bp that are absolutely conserved (100% homology with no insertions or deletions) in human, mouse, and rat genomes [2]. Many of these elements possess tissue-specific enhancer activity [3-5] and others have been shown to associate with splicing regulators. Evolutionary conservation has become a powerful tool to identify functionally important regions in the human genome [6]. A high proportion of UCRs show extreme conservation within mammals only and nearly 47% of UCRs in human have been localized to exons of genes involved in RNA processing or in the regulation of transcription and development [2,7]. The reasons for this extreme conservation remain a mystery, but it was proposed that UCRs play a role in the ontogeny and phylogeny of mammals and other vertebrates. This idea is supported by the identification of a distal enhancer and an ultra-conserved exon derived from a retroposon active more than 400 million years ago in lobe-finned fishes and terrestrial vertebrates, and maintained as active in the 'living fossil' coelacanth [2]. Another study showed the concurrent presence of enhancer and transcript functions in non-exonic UCRs, and suggested that they may belong to non-coding RNAs (ncRNAs) [8]. Recently, a positive correlation of expression with conservation and epigenetic marks was described in T-UCRs, among other ncRNAs [9]. Despite still having largely unknown roles, T-UCRs are thus now thought to act as 'regulators' of other RNAs [10].

Recent studies suggested that UCRs could contribute to the development of malignancies [11,12]. Genome-wide profiling revealed that UCRs have distinct signatures in human leukemias and carcinomas [13] and are frequently located at fragile sites and in cancer-associated genomic regions [14]. Clinical findings also suggested that UCR signatures can have independent prognostic value in high-risk neuroblastoma patients [15] by providing additional prognostic value in conjunction with *N-MYC* activity/amplification [16]. Additionally, SNPs within UCRs were associated with increased familial breast cancer risk [17].

We performed this large scale study to identify UCR activity in cancer, analyzing almost 900 human samples from a panel of 46 normal tissues and 10 solid cancers using a custom-made microarray platform.

## Methods

### UCR expression arrays

We studied the expression of UCRs using the Ohio State University Comprehensive Cancer Center (OSUCCC) custom microarray [18]. The Gene Expression Omnibus describes the OSU-CCC 4.0 platform under accession number GPL14184. Briefly, a sense and an anti-sense 40-mer probe were designed for each of 481 UCRs. Each probe was printed in duplicate in two different slide locations, and therefore quadruplicate measures were available. Total RNA (2 μg) were used for labeling and hybridization. The microarrays were hybridized in 6X SSPE (0.9 M NaCl/60 mM NaH_2_PO_4_ · H_2_O/8 mM EDTA, pH 7.4)/30% formamide at 25°C for 18 h, washed in 0.75X TNT (Tris HCl/NaCl/Tween 20) at 37°C for 40 minutes. Processed slides were scanned using a microarray scanner (Axon Molecular Devices, Sunnyvale, CA, USA), with the laser set to 635 nm, at a fixed PMT (photomultiplier tube) setting, and a scan resolution of 10 mm. Microarray images were analyzed using GenePix Pro and post-processing was performed essentially as described earlier [18].

### Data analysis

T-UCRs were retained when present in at least 20% of samples and when at least 20% of them had a fold change of more than 1.5 from the gene median. Absent calls were thresholded prior to normalization and statistical analysis. Normalization was performed by using quantiles [19]. First, all samples were classified according to organ, tissue and cell type; the samples were then grouped in systems (Additional file 1) and cancer types (Additional file 2). To assess the specificity of UCR expression across groups we used information content (IC) [20]. Differentially expressed RNAs were identified using *t*-tests over two-class experiments or *F*-tests over multiple classes (that is, various normal tissues) within the class comparison tool [21].

### Computational methods and folding free energies

Computational methods were used to investigate uc.283-plus and predict secondary structure for its RNA sequence. To investigate secondary structure, we used the RNAfold web server in the freely available ViennaRNA package version 2.0 to compute the centroid secondary structure and the corresponding free energy changes for folding, the minimum free energy (MFE; kcal/mol). In order to detect putative microRNA (miRNA) target sites in the considered region, we used IntaRNA, freely available online [22,23]. Calculation of accessibility is based on ensemble free energies. Ensemble free energies were calculated using a partition function approach assuming global folding of the ncRNA and local folding of the mRNA. For this purpose, RNAfold and RNAup are integrated into IntaRNA via the ViennaRNA library [24]. Highly stable miRNA-target duplexes are represented as having a very low hybridization energy.

### RNA expression and validation

Mouse embryonic stem cell (ESC) lines (V6.4; hybrid 129/C57Bl/6) were cultured at 37°C in 5% CO_2_ in specific medium and total RNA was extracted using TRIzol. RNA samples were treated with 1 U DNase I Ampl Grade (Invitrogen Life Technology, Carlsbad, CA, USA) to remove any contaminating genomic DNA.

Total RNA was reverse transcribed using random hexamers (Invitrogen Life Technologies, Carlsbad, CA, USA). T-UCR expression was quantified by quantitative PCR (qPCR) with SYBR green. All reactions were performed in triplicate and 18S rRNA was used as reference. The triplicate Ct values were averaged and normalized Ct (∆Ct) calculated. The living conditions of the mice were appropriate for their species and all mouse experiments were approved by the Institutional Animal Care and Use Committee (IACUC) and University Laboratory Animal Resources (ULAR) of The Ohio State University. The animals were euthanized as per the IACUC approved guidelines and protocols before harvesting the tissues. RNA purity was assessed by the ratio of absorbance at 260 and 280 nm (A260/280 nm) using a NanoDrop ND-1000 (NanoDrop Inc., Wilmington, DE, USA). All tissues were obtained under the guidelines of approved protocols from the Ohio State University Internal Review Board and informed consent was obtained from each subject. RNA profiles for the human tissue and cell line samples have been deposited at ArrayExpress (E-TABM-969 and E-TABM-970) and at the NCBI Gene Expression Omnibus (GSE16654). Cancers samples were deposited at ArrayExpress (E-TABM-971 for breast carcinoma, E-TABM-46 for colorectal adenocarcinoma, E-TABM-22 for lung cancer, E-TABM-343 for ovarian carcinoma, and E-TABM-49 for prostate adenocarcinoma), and at the Gene Expression Omnibus (GSE7828 for colorectal carcinoma, GSE20099 and GSE24839 for esophagus carcinoma, GSE53504 for glioma and GSE14936 for lung adenocarcinoma and squamous carcinoma).

## Results and discussion

### T-UCR expression in normal human tissues

**Figure 1:**
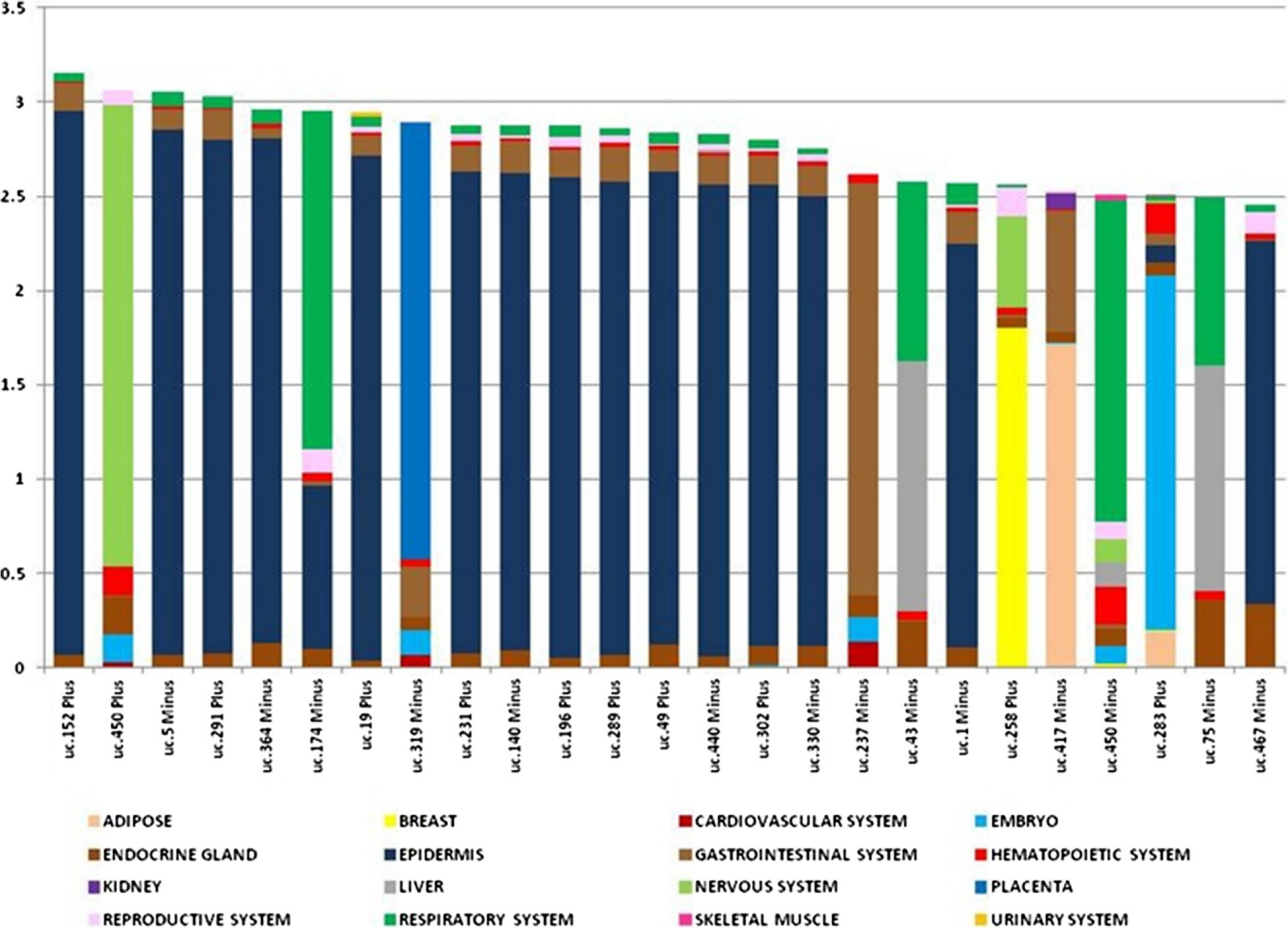
**Distinct T-UCR signatures in different clusters of normal tissues (information content standardization).** T-UCR selectivity in 46 normal tissues grouped by 16 systems. The tissue selectivity was calculated by using the information content (IC), shown on the y-axis; each color represents a system. The most represented cluster was the epidermis; 15 T-UCRs showed strong differential expression (IC >2) for this cluster.

We tested the expression of UCRs in 374 samples from 46 types of normal tissues, belonging to 16 histological groups [25]. For UCR profiling we used the OSUMC microarray platform, previously validated in two large scale studies [18,26]. This platform has probes for 481 putative T-UCRs in either genomic strand (designated ‘plus’ or ‘minus’; in some other studies defined ‘+’ or ‘+A’, respectively). A global analysis showed that only a portion of the UCRs (296 out of 962) was expressed in human tissues (*P*-value <0.001; Additional file 3); 48% of these were non-exonic, 26% putative exonic and 26% exonic, proportions that reflect previously published studies [13,14]. Fifty-seven T-UCRs were transcribed bi-directionally (Additional file 4). Tissue selectivity was calculated using the IC [20]. The most tissue-selective UCRs were represented by a group of 15 UCRs, mostly expressed in epidermis, with an IC value ranging from 3.23 to 1.70 (Figure 1; Additional file 5). These 15 T-UCRs were not located in the same genomic cluster. Another tissue-specific RNA was uc.450-plus, which is highly expressed in the central nervous system. This finding confirms previous data showing that uc.450 was expressed in the dorsal root ganglion and the neural tube [27]. In particular, Visel *et al*. [27] hypothesized an enhancer function for hs385, which fully includes uc.450. Interestingly, the opposite probe, uc.450-minus, was specific for the respiratory system. Uc.174-minus, located on the opposite strand of the *MATR3* exon, was expressed mainly in the respiratory system and epidermis. The placenta was characterized by the expression of uc.319-minus (intergenic), while uc.237-minus (intronic) was restricted to the gastrointestinal system. Uc.43-minus (intronic; our probe is homologous to *Mus musculus* CN668140 EST), uc.75-minus, located on the opposite strand of the last exon of *ZEB2*, and uc.42-plus (intronic) were expressed in both liver and the respiratory system. In addition, uc.417-minus was expressed in adipose tissue and in the gastrointestinal system. The only UCR selective for embryo was uc.283-plus, on which we focus for the rest of this study.

### A single T-UCR is differentially expressed in pluripotent stem cells

**Figure 2:**
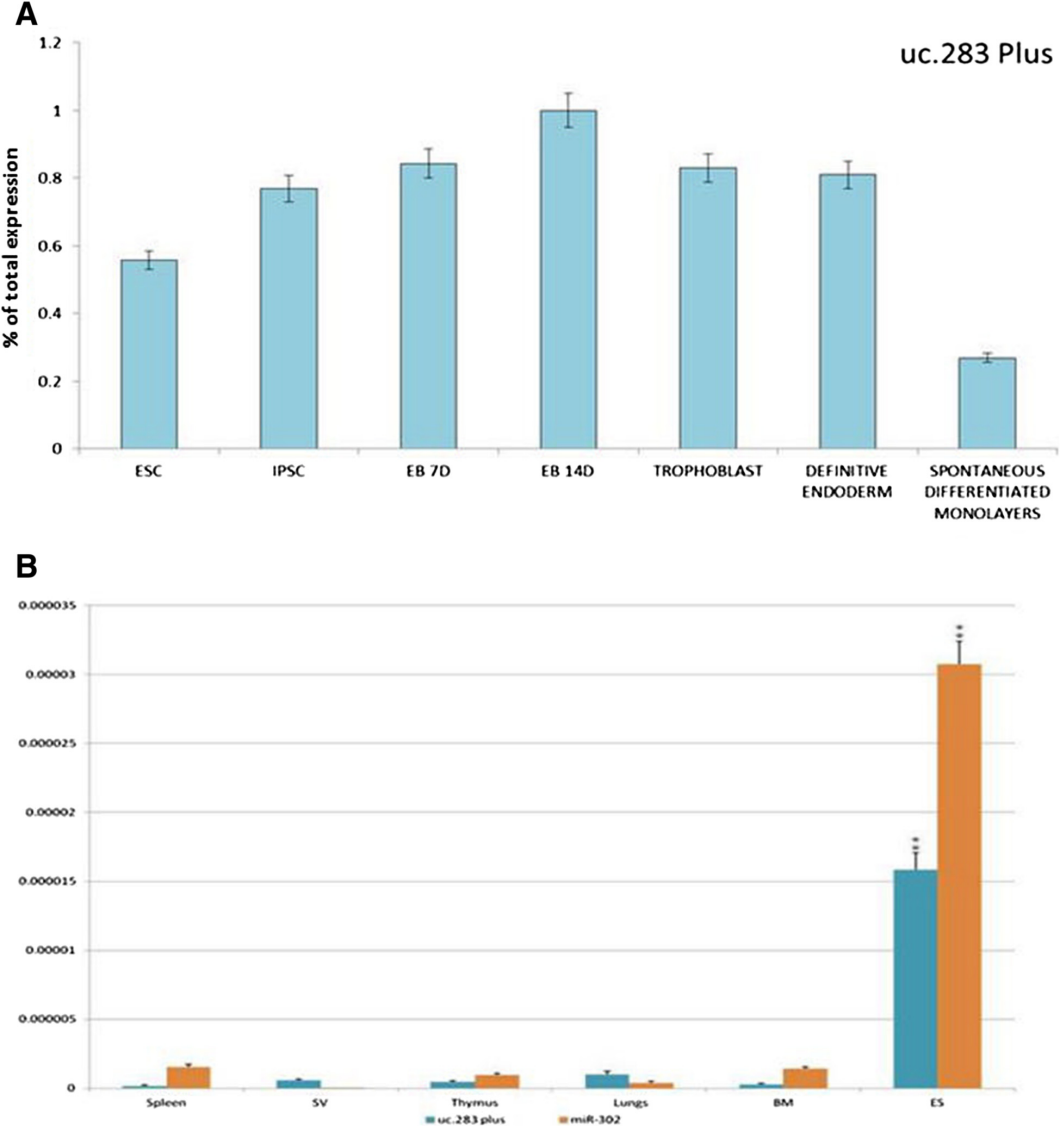
**Expression levels of uc.283-plus in embryonic stages and adult tissues. (A)** Percentage of total uc.283-plus expression in seven different embryonic tissues: ESCs, induced pluripotent stem cells (IPSCs), 7 day and 14 day embryoid bodies (EB7 and EB14), trophoblasts, definitive endoderm and spontaneously differentiating monolayers. **(B)** Real-time PCR confirms the results of the microarray analysis. Uc.283-plus is expressed at higher levels in mouse ESCs (ES) than in adult tissues, such as spleen, seminal vesicles (SV), thymus, lungs and bone marrow (BM). miR-302 was used as a positive control. Error bars represent standard deviation. **P-values <0.01.

Our extensive study of UCR tissue selectivity identified only one RNA specific for the embryo. To further investigate this finding we studied ESCs and induced pluripotent stem cells. Uc.283-plus, a 277 nucleotide-long sequence located at chr10:50,604,757-50,605,033, was sufficient to discriminate between adult tissues and pluripotent stem cells. Figure 2A shows the expression of uc.283-plus at distinct stages of differentiation. Uc.283-plus displayed high values in human ESCs and induced pluripotent stem cells, increased in trophoblasts at 7 and 14 days of embryoid body differentiation, and decreased in definitive endoderm to reach the lowest values in spontaneously differentiated monolayers.

To validate our microarray data that suggest uc.283-plus is pluripotent specific, we used qPCR on RNAs extracted from the mouse ESCs and RNAs from five mouse adult tissues (spleen, seminal vesicles, thymus, lung, and bone marrow). We used ESC-specific miR-302 as a positive ncRNA control [20,25]. The qPCR (Figure 2B) confirmed strong and mouse ESC-selective expression for both miR-302 and uc.283-plus (Wilcoxon test *P*-value <0.01) (Additional file 6). No enhancer activity was associated in Vista Enhancer Browser [5] with uc.283. In a further quest to investigate the possible function of uc.283, we analyzed a 2,000 bp region surrounding this UCR using the UCSC Genome Browser. In the H1-hESC chromatin state segmentation (HMM track from ENCODE/Broad), this sequence might be an inactive or poised promoter [28]. In addition, the edges of uc.283 overlap with the initial tracts of two open chromatin regions (OpenChrom_15681 and OpenChrom_15682). Data from the methyl 450 K bead array track (ENCODE/HAIB) showed that, in close proximity to the start of uc.283-plus, the CpG sequence was not methylated in H1-hESC and human umbilical vein endothelial cells, and partially or totally methylated in other cell types. These findings, together with the proximity to open chromatin regions, suggested that uc.283-plus could be located in a euchromatic region during embryonic development and in ESCs. Furthermore, uc.283-plus is not present in the catalogue of lncRNAs originated from divergent transcription at promoters of active protein-coding genes [29]. In particular, there seems to be no connection between uc.283-plus and the adjacent CpG island, containing the DRGX (Dorsal root ganglia homeobox) promoter (on the opposite strand).

### Among solid cancers uc.283-plus is mostly expressed in glioma

**Figure 3:**
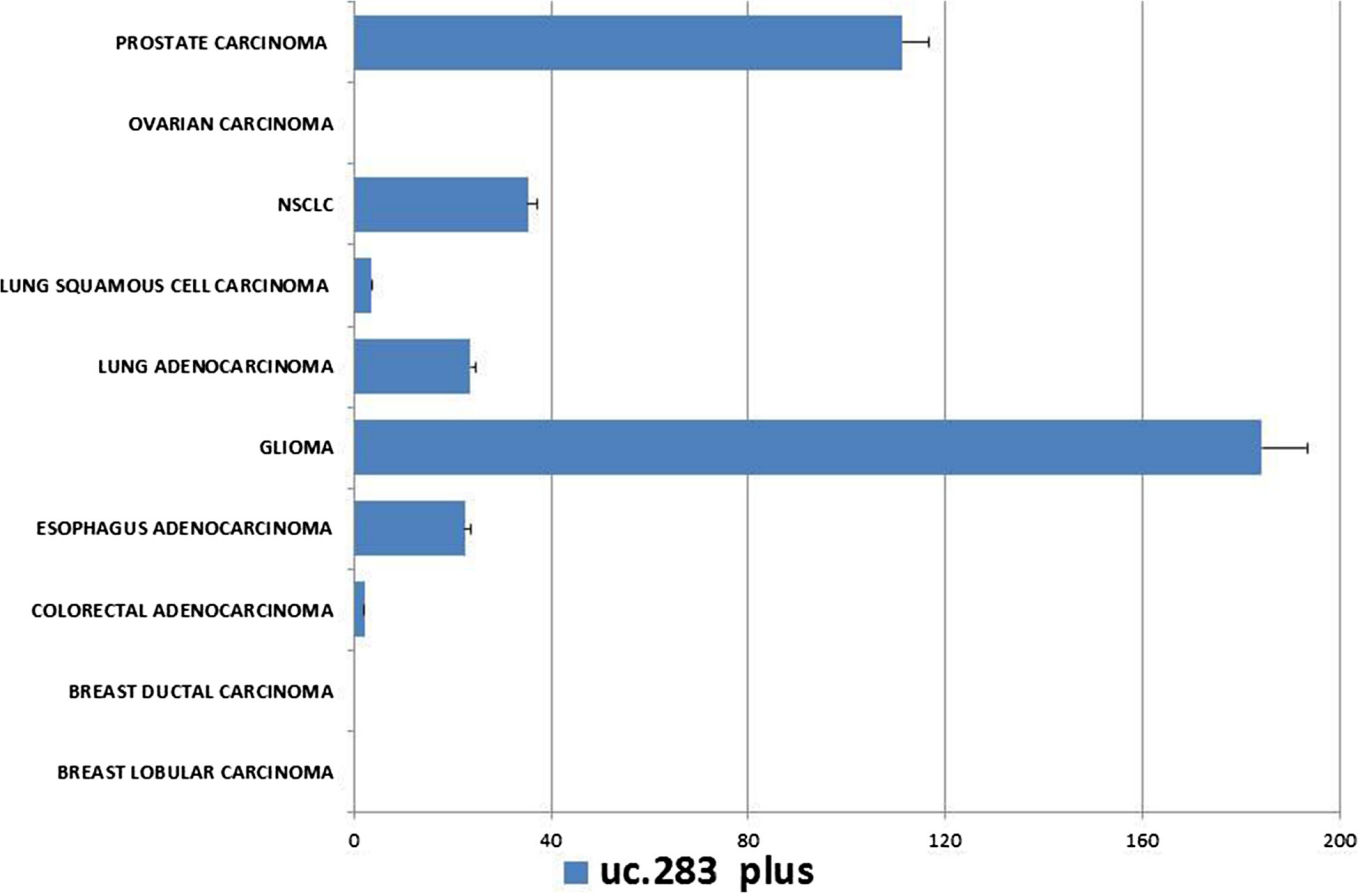
**Uc.283-plus expression in solid cancers based on 510 samples.** The highest expression was in glioma, followed by prostate adenocarcinoma. No expression was detected in breast lobular and ductal carcinoma and in ovarian cancers. NSCLC, non-small-cell lung carcinoma. Error bars represent standard deviation.

Cancer stem cells are a highly debated issue in oncology [30,31]. Since uc.283 was associated with pluripotency, we assessed its RNA levels in about 500 tumors from several types of solid cancers. Surprisingly, uc.283-plus was over-expressed in prostate adenocarcinoma and glioma samples (Figure 3). Recently, lncRNAs were investigated in glioma to define grade and histological differentiation of the tumor [32]. The highest levels of expression were found in glioma, considered one of the most aggressive cancers with high propensity for proliferation and tissue invasion. It is tempting to speculate that the high expression of uc.283-plus in glioma is correlated with a 'cancer stem cell phenotype', a well-studied event occurring in glioma [33]. Interestingly, Lujambio *et al*. [34] identified an RNA in the uc.283 genomic region but transcribed from the opposite strand in various types of cancer cell lines. They also showed that uc.283-minus undergoes specific CpG island hypermethylation, suggesting that it could be regulated by epigenetic alteration. Recently, Hudson *et al*. [35] confirmed the up-regulation of uc.283-minus in a prostate cancer cell line treated with the DNA hypomethylating agent 5-azacytidine and with the histone deacetylase inhibitor trichostatin A. Hudson *et al*. produced a list of all the possible ucRNA-mRNA interactions based on sequence complementarity according to the thermodynamics of the loop-loop RNA interactions [36,37]. In order to assess whether uc.283-plus has a functional role, we checked for possible interactions with the mRNAs listed but did not find any. Subsequently, we hypothesized that it has a 'sponge function' for recruitment of miRNAs or other class of ncRNAs. We examined the possible secondary structure of uc.283-plus (Figure S1A,B in Additional file 7) [24] and submitted the RNA sequence to the web-tool application RegRNA2.0 [38], analyzing predicted miRNA target sites with a score ≥150 and a free energy of -20 or less. We found that our transcript sequence could be a target of three miRNAs: hsa-miR-455-5p, has-miR-640 and has-miR-1909-3p. We verified these possible interactions using another algorithm, IntaRNA (Figure S1C in Additional file 7) [22]. Interestingly, hsa-miR-1909-3p was discovered in human ESCs by deep sequencing of small RNA libraries [39] and targeted genes, such as *DICER1*, *SOX2* and *NOTCH1* [40]. Hsa-miR-455-5p and hsa-miR-640 were deregulated in several cancers but were not the most abundant isoforms and they have not been well characterized yet [41,42]. Very recently, Liz *et al*. [10] showed that the long ncRNA uc.283-minus controlled pri-miRNA processing. This ncRNA-ncRNA interaction prevents pri-miRNA-195 cleavage by Drosha. Therefore, understanding the interactions of this kind of ncRNA is of particular importance to pinpoint their biological meaning.

## Conclusion

We report here the genome-wide analysis of UCR tissue selectivity among 16 human histologic groups, corresponding to 46 different normal tissues. A fraction of UCRs were tissue-selective, while others were broadly expressed. Uc.283-plus was highly expressed in pluripotent ESCs and induced pluripotent stem cells. Uc.283-plus may play an important role in pluripotency and among solid cancers it is highly expressed in glioma. The understanding of the biological roles of UCRs, as those of the other lncRNAs, remains an open challenge. This study can be a starting point for the further characterization of UCR activities in normal and cancer tissues.

## Additional files

Additional file 1: Table S1. The number of microarray chip samples used is reported for each system type. Additional file 2: Table S2. The number of microarray chip samples used is reported for each cancer type. Additional file 3: Table S3. Class comparison analysis: expression levels of tissue-selective T-UCRs. Additional file 4: Table S4. T-UCR that are bidirectional in normal cluster tissues. Additional file 5: Table S5. Tissue specific T-UCRs in normal tissues. Sorted by information content (IC). The genomic coordinates came from hg19. Additional file 6: Table S6. Expression levels are calculated as 2^(-ΔΔCT)^ using 18S rRNA as a reference. Average of at least three experiments, each PCR performed in triplicate. Standard errors (ERR.ST.) are reported. Additional file 7: Figure S1.
**(A)** The uc.283-plus folding structure for the minimum free energy prediction. The optimal secondary structure has a minimum free energy of -65.70 kcal/mol. **(B)** The centroid secondary structure with a minimum free energy of -32.80 kcal/mol. **(C)** Hsa-miR-640, hsa-miR-1909-3p and hsa-miR-455-4p target uc.283-plus in three different positions with different hybridization energy values.

## Abbreviations

bp: base pair
ESC: embryonic stem cell
IC: information content
lncRNA: long non-coding RNA
miRNA: microRNA
ncRNA: non-coding RNA
qPCR: quantitative polymerase chain reaction
SNP: single-nucleotide polymorphism
T-UCR: transcribed ultra-conserved region
UCR: ultra-conserved region
