# Supplementary figures and images for: A large scale expression study associates uc.283-plus lncRNA with pluripotent stem cells and human glioma
Source: Genome Med. 2014 Oct 2;6(10):76. doi: 10.1186/s13073-014-0076-4 (PMC4210590; doi:10.1186/s13073-014-0076-4)

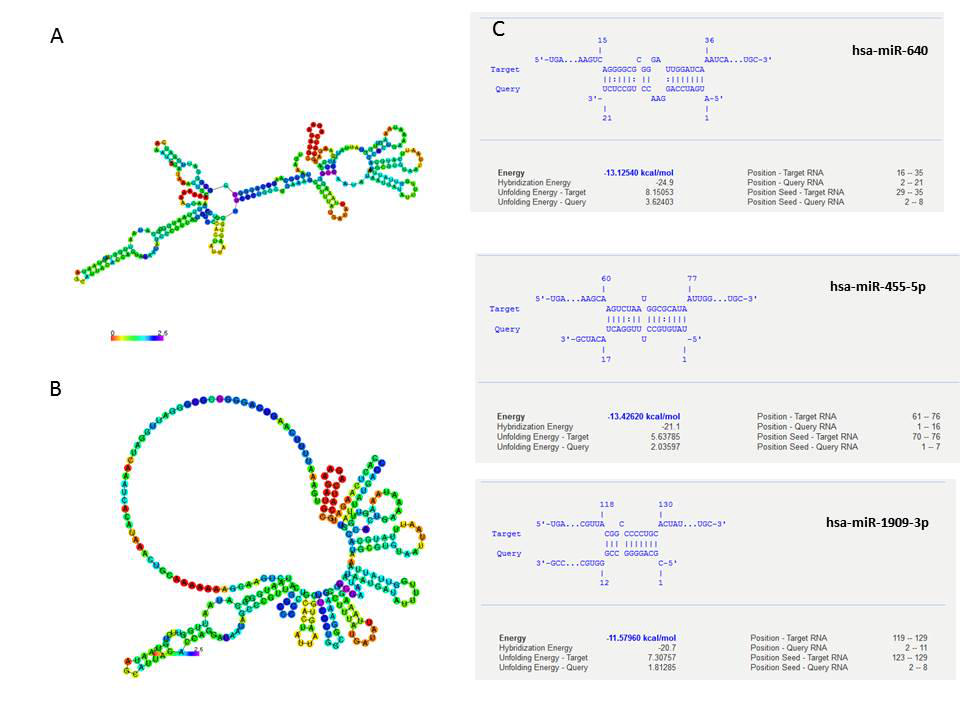

Supplement: Additional file 7: Figure S1. — (A) The uc.283-plus folding structure for the minimum free energy prediction. The optimal secondary structure has a minimum free energy of -65.70 kcal/mol. (B) The centroid secondary structure with a minimum free energy of -32.80 kcal/mol. (C) Hsa-miR-640, hsa-miR-1909-3p and hsa-miR-455-4p target uc.283-plus in three different positions with different hybridization energy values. [file 13073_2014_76_MOESM7_ESM.tiff]
